# Design and Rationale of the National Observational Multicentric Tunisian Registry of Hypertension: Protocol for Evaluating Hypertensive Patient Care in Clinical Practice

**DOI:** 10.2196/21878

**Published:** 2022-09-02

**Authors:** Leila Abid, Rania Hammami, Salem Abdesselem, Selim Boudiche, Ben Slima Hédi, Khaled Sayahi, Amine Bahloul, Ikram Chamtouri, Salma Charfeddine, Lamia Rais, Meriem Drissa, Badreddine Ben Kaab, Hassen Ibn hadj amor, Lilia Ben Fatma, Riadh Garbaa, Sabrine Boukhris, Allouche Emna, Manel Ben Halima, Nesrine Amdouni, Shayma Ghorbel, Sabrine Soudani, Imen Khaled, Syrine Triki, Feten Bouazizi, Imen Jemai, Ouday Abdeljalil, Yemna Ammar, Amani Farah, Adnen Neji, Zeineb Oumaya, Sana Seghaier, Samir Mokrani, Hamza Thawaba, Hela Sarray, Khalil Ouaghlani, Houssem Thabet, Zeineb Mnif, Boujelben Masmoudi Fatma, Mohamed Sghaier, Roueida Khalifa, Sami Fourati, Yassmine Kammoun, Syrine Abid, Chiheb Hamza, Syrine Ben Jeddou, Lassaad Sabbah, Rim Lakhdhar, Najla Dammak, Tarak Sellami, Basma Herbegue, Alia Koubaa, Faten Triki, Tarek Ellouze, Aicha Hmoudi, Ikhlas Ben Ameur, Mohamed Mongi Boukhchina, Neila Abid, Wejdene Ouechtati, Nizar Nasrallah, Yousra Houidi, Fathia Mghaieth Zghal, Ghodhbane Elhem, Mounira Chayeb, Chenik Sarra, Samira Kaabachi, Nizar Saadaoui, Ines Ben Ameur, Moufida Affes, Sana Ouali, Mouna Chaker, Hela Naana, Dghim Meriem, Mourad Jarrar, Jihen Mnif, Ahmed Turki, Ihsen Zairi, Jamel Langar, Safa Dardouri, Imen Hachaichi, Rafik Chettaoui, Wajih Smat, Amel Chakroun, Khadija Mzoughi, Rachid Mechmeche, Afef Ben Halima, Sahar Ben Kahla Koubaa, Slim Chtourou, Maalej Mohamed abdelkader, Mohsen Ayari, Moufid Hadrich, Tlili Rami, Fares Azaiez, Imen Bouhlel, Samir Sahnoun, Habib Jerbi, Ben Mrad Imtinene, Leila Riahi, Mohamed Sahnoun, Abdelhamid Ben Jemaa, Amal Ben Salem, Bassem Rekik, Maroua Ben Doudou, Mohamed Rachid Boujnah, Anissa Joulak, Abid Omar, Rabie Razgallah, Milouchi Sami, Elyes Neffati, Habib Gamra, Soraya Ben Youssef, Wissem Sdiri, Nejeh Ben Halima, Youssef Ben Ameur, Salem Kachboura, Sondes Kraiem, Wafa Fehri, Lilia Zakhama, leila Bezdah, Mourali Mohamed Sami, Habiba Drissa, Mohamed Faouzi Maatouk, Samir Kammoun, Faouzi Addad

**Affiliations:** 1 Tunisian Society of Cardiology and Cardiovascular Surgery Tunis Tunisia; 2 Cardiology Department, Hedi Chaker-Sfax University Hospital Faculty of Medecine of Sfax University of Sfax Sfax Tunisia; 3 Cardiology Department, La Rabta 1 (Pr Mourali) University Hospital Faculty of Medecine of Tunis University of Tunis Tunis Tunisia; 4 Cardiology Department, Hospital of Menzel Bourguiba Bizerte Tunisia; 5 Cardiology Department, ElKef Hospital Elkef Tunisia; 6 Cardiology Department B, Fattouma Bourguiba University Hospital Faculty of Medecine of Monastir University of Monastir Monastir Tunisia; 7 Nephrology Department, La Rabta University Hospital Faculty of Medecine of Tunis University of Tunis Tunis Tunisia; 8 Cardiology Department, La Rabta 2 (Pr Drissa) University Hospital Faculty of Medecine of Tunis University of Tunis Tunis Tunisia; 9 Cardiology Department, Tahar Sfar Hospital Mahdia Tunisia; 10 Cardiology Department, Charles Nicole University Hospital Faculty of Medecine of Tunis University of Tunis Tunis Tunisia; 11 Cardiology Department, Habib Bourguiba Hospital Medenine Tunisia; 12 Basic Health Centers Medenine Tunisia; 13 Habib Bourguiba Hospital Medenine Tunisia; 14 Hospital of Tozeur Tozeur Tunisia; 15 Private clinic Tunis Tunisia; 16 Hospital of Mateur Bizerte Tunisia; 17 Cardiology Department, Farhat Hached Hospital Faculty of Medecine of Sousse University of Sousse Sousse Tunisia; 18 National Social Security Fund Sfax Tunisia; 19 Private clinic Sfax Tunisia; 20 Nephrology Department, Hedi Chaker-Sfax University Hospital Faculty of Medecine of Sfax University of Sfax Sfax Tunisia; 21 Basic Health Centers Ben Arous Tunisia; 22 Basic Health Centers Tataouine Tunisia; 23 Private clinic Gabes Tunisia; 24 Private clinic Ariana Tunisia; 25 Basic Health Centers Tunis Tunisia; 26 Cardiology Department, The Main Military Instruction Hospital of Tunis Faculty of Medecine of Tunis University of Tunis Tunis Tunisia; 27 Cardiology Department, Habib Thameur Hospital Faculty of Medecine of Tunis University of Tunis Tunis Tunisia; 28 Basic Health Centers Gabes Tunisia; 29 Hospital of Habib Bougatfa Bizerte Tunisia; 30 Cardiology Department, Abderrahmen Mami-Ariana Hospital Faculty of Medecine of Tunis University of Tunis Ariana Tunisia; 31 Cardiology Department, Mahres Hospital Faculty of Medecine of Sfax University of Sfax Sfax Tunisia; 32 Private clinic Nabeul Tunisia; 33 Cardiology Department, Mongi Slim Hospital Faculty of Medecine of Tunis University of Tunis Tunis Tunisia; 34 Private clinic Sousse Tunisia; 35 Dacima Tunis Tunisia; 36 Cardiology Department, University Hospital Sahloul Faculty of Medecine of Sousse Sousse Tunisia; 37 Cardiology Department A, Fattouma Bourguiba University Hospital Faculty of Medecine of Monastir University of Monastir Monastir Tunisia; 38 Cardiology Department, Internal Security Forces Hospital Faculty of Medecine of Tunis University of Tunis Tunis Tunisia; 39 Cardiology Department, Bougatfa Hospital Bizerte Tunisia; 40 Cardiology Department, Ibn El Jazzar Hospital Kairouan Tunisia

**Keywords:** National Tunisian Registry, hypertension

## Abstract

**Background:**

This study was designed to evaluate the care of hypertensive patients in daily clinical practice in public and private centers in all Tunisian regions.

**Objective:**

This study will provide us an overview of hypertension (HTN) management in Tunisia and the degree of adherence of practitioners to international recommendations.

**Methods:**

This is a national observational cross-sectional multicenter study that will include patients older than 18 years with HTN for a duration of 4 weeks, managed in the public sector from primary and secondary care centers as well as patients managed in the private sector. Every participating patient signed a consent form. The study will exclude patients undergoing dialysis. The parameters that will be evaluated are demographic and anthropometric data, lifestyle habits, blood pressure levels, lipid profiles, treatment, and adherence to treatment. The data are collected via the web interface in the Dacima Clinical Suite.

**Results:**

The study began on April 15, 2019 and ended on May 15, 2019. During this period, we included 25,890 patients with HTN. Data collection involved 321 investigators from 24 Tunisian districts. The investigators were doctors working in the private and public sectors.

**Conclusions:**

Observational studies are extremely useful in improving the management of HTN in developing countries.

**Trial Registration:**

ClinicalTrials.gov NCT04013503; https://clinicaltrials.gov/ct2/show/NCT04013503

**International Registered Report Identifier (IRRID):**

DERR1-10.2196/21878

## Introduction

Hypertension (HTN) is widespread in many developing and developed countries [1]. According to a 2010 report by the Institute of Medicine, HTN is a neglected disease, often ignored by the general public and underestimated by the medical world [2].

However, more than a quarter of the world's adult population is already hypertensive, and this number is expected to increase to 1.56 billion by 2025 [3,4]. Unfortunately, HTN causes more than 7 million premature deaths a year and contributes to 4.5% of the global disease burden [5,6]. HTN is also a risk factor for cardiovascular diseases responsible for 30% of all deaths worldwide, which can be controlled [7,8].

Many clinical trials have shown that stringent blood pressure (BP) control can significantly reduce cardiovascular risk [9,10] and certain sequelae such as stroke, myocardial infarction, sudden cardiac arrest, peripheral vascular disease, and renal insufficiency [11-13].

HTN in Tunisia is a public health issue considering its current frequency [14-16]. As no relevant updated data exist, this work presents a new relational database to obtain an overview of the management of hypertensive patients in Tunisia and enable adequate protection planning. Therefore, a multicenter observatory focusing on the demographic, anthropometric, and therapeutic features of HTN in Tunisia is mandatory. The collected data will allow us to assimilate our practices and know the degree of adherence by practitioners to international recommendations for this pathology treatment.

The aim of the National Tunisian Registry of Hypertension (NATURE-HTN) is to describe the epidemiological profile of HTN in Tunisia, determine the cardiovascular risk level of Tunisian hypertensive patients, and obtain an idea about the percentage of patients treated for therapeutic purposes.

## Methods

Several secondary end points are defined, such as evaluating the therapeutic adequacy with respect to that specified in the international recommendations of the European Society of Hypertension-European Society of Cardiology (ESH-ESC) 2018 for managing HTN and evaluating the degree of therapeutic inertia.

### Study Design and Patient Enrollment

A national observatory, longitudinal, and multicentric register study was carried out over a month, without clinical follow-up and investigations. We included patients managed in the private and public sectors as well as from primary and secondary care centers.

During office visits, we included patients above 18 years with known or newly diagnosed elevated BP after signing a consent form. Except for severe HT (eg, grade 3 and especially high-risk patients), the new HT diagnosis was confirmed according to the ESC and ESH guidelines as either out-of-office BP measurements above the recommended thresholds or repeated office BP measurements above 140 mmHg for the systolic blood pressure (SBP) and 90 mmHg for the diastolic blood pressure (DBP) during more than 1 visit [11].

#### Exclusion Criteria

We excluded patients undergoing hemodialysis, pregnant women, individuals classified as white-coat HT patients, and patients who refused to sign the consent form from the study.

During the office visit, the physician had to complete the case report form of the registry after patient interrogation and examination. Information on sociodemographic characteristics including age, gender, education level, health insurance, smoking, diabetes, pulmonary diseases, hypothyroidism, moderate renal failure history defined by an MDRD (Modification of Diet in Renal Disease) creatinine clearance <60 mL/min, coronary disease, and history of stroke were collected.

The interview included questions related to drug compliance and salt intake as well as sport practice. Physical activity was considered regular when it was performed at least 30 minutes 3 times a week. During physical examination, we measured the weight and height to assess the BMI (BMI= weight/[height]^2^). Obesity is operationally defined as a BMI exceeding 30 kg/m^2^ and is subclassified into moderate (BMI=30-34.9), morbid (BMI=35-39.9), and severe (BMI≥40). BP measurements were conducted using a standardized auscultatory or oscillometric sphygmomanometer after at least 15 min of rest. We recorded 2 separate measurements at least 3 minutes apart and considered the mean of the 2 measurements. In patients with asymmetric BP between the 2 arms, we considered the higher pressure.

We confirmed whether the patients had a sinus rhythm or atrial fibrillation through electrocardiograms and searched for left ventricle hypertrophy (LVH) based on the definition recommended by the ESC/ESH guidelines (Sokolow-Lyon index>35 mm or R in a VL≥11 mm) [11]. We searched for LVH in echocardiographic findings as well (if the patients underwent echocardiography during the last year).

We also recorded the biological tests performed during the last 6 months before the office visit, especially creatinine, glycaemia, cholesterol, kaliemia, and microalbuminuria (if performed during the last year).

To assess and control BP, we evaluated only patients diagnosed with HTN for more than 6 months. The primary end point in our study was the rate of HTN control. Uncontrolled HTN was defined according to the ESC/ESH guidelines as an average SBP above 140 mmHg and an average DBP above 90 mmHg [11].

#### Ethics Consideration

The study protocol, the consent form prepared by the steering committee, and the creation of the registry were approved by the ethic committee of the Hospital of the Internal Security Forces. Patients must give their consent before being included in the registry.The steering committee will check for any violation of the protocol by the investigators (inclusion of patients who are not eligible according to selection criteria) and will make a decision on the exclusion of the concerned patient(s).

### Sample Size and Data Collection

Eligible patients according to the inclusion and exclusion criteria were selected by more than 600 investigators (working in the public and private sectors) specialized in cardiology, nephrology, endocrinology, internal medicine, and general medicine.

The target simple size of the whole study was calculated using the following formula: N = Z^2^ × p0 × (1 – p0)/i^2^ (Z=1.96 if we consider a confidence level of 95%, and p0=24.1%; it corresponds to the control rate of HTN in the TAHINA project). More than 7000 patients were needed.

**Figure 1:**
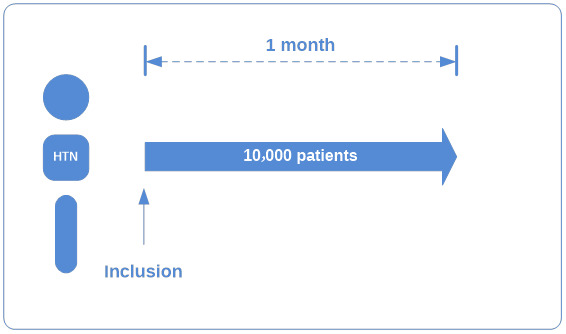
Protocol of population inclusion. HTN: hypertension.

Patients were included continuously until the end of the inclusion period. The inclusion lasted for 1 month without any follow-up, as shown in Figure 1. Given the observational nature of the NATURE-HTN study, no specific treatment or intervention is planned for HTN management. Patients should be cared for according to their usual medical habits.

The data were collected via the web interface in the Dacima Clinical Suite (Dacima Software Inc). The electronic data capture platform complies with the following international standards: Food and Drug Administration 21 Code of Federal Regulations part 11, Health Insurance Portability and Accountability Act, and International Conference on Harmonization.

### Statistical Analysis

The Dacima Clinical Suite platform enables the collection of data through the web interface and their extraction in the SAS or SPSS format. The statistical analysis is exploratory, involving the calculation of the 95% CI.

The data will be described for the entire population of interest. Statistical tests will be bilateral with a statistical significance threshold of 5%. ANOVA will be performed for the quantitative variables according to their normal distribution (parametric tests for the variables that follow a normal distribution and nonparametric tests in the other cases). A chi-square test will be performed for the categorical variables (or corrected for continuity if the validity conditions for the chi-square test are not met, namely if the theoretical number is less than 5).

The data review committee is composed of the coordinators of the steering committee of the study as well independent experts in data management. The purpose of the data review committee is to manage data queries and validate the final statistical analysis plan. The committee will also be responsible for validating subsequent publications that will be conducted.

The protocol of the NATURE-HTN registry has been approved by the Tunisian Society of Cardiology and Cardiovascular Surgery. The NATURE-HTN study has been submitted to ClinicalTrials.gov and registered under the identifier NCT04013503.

The NATURE-HTN registry does not impose any specific intervention. Treatments of the patients follow the usual recommendations for managing HTN. Clinical events occurring during the study are not to be recorded.

### Study Sponsorship

The NATURE-HTN registry study is sponsored by the Tunisian Society of Cardiology and Cardiovascular Surgery.

## Results

The study began on April 15, 2019 and ended on May 15, 2019. During this period, we included 25,890 patients with HTN. Data were collected by 321 investigators from 24 Tunisian districts. The investigators were doctors working in the private and public sectors. Cardiologists included 71% (n=18,382) of the patients, and general practitioners included approximately 25% (n=6473) of the patients.

## Discussion

### Study Significance

NATURE-HTN is the largest observational registry in the field of HTN management in Tunisia. The results will be compared to those of the TAHINA study. The latter was a national survey including 8007 patients aged between 35 and 70 years. The prevalence of hypertension was 30.6%, and it was higher in women (n=2682, 33.5%) than in men (n=2186, 27.3%). Only 38.8% (n=3107) of those with HTN were aware of their diagnosis, of which 84.8% (2635/3107) were receiving treatment. BP control was achieved in only 24.1% (635/2635) of the treated hypertensive patients. Women were more aware than men (1202/2682, 44.8% vs 630/2186, 28.8%), but the rates of treatment and control of HTN did not differ between the 2 genders. Higher age, being female, lower education level, and urban area emerged as important correlates of HTN awareness [15]. Certainly, there is an urgent need for comprehensive integrated population-based intervention programs to ameliorate the growing problem of HTN in Tunisians. BP control has not improved even in the developed countries. In France, according to the Flash studies, the percentage of the participants with BP is approximately 51%, and the use of monotherapy is one of the major problems registered in the French survey [17].

### Conclusions

BP control should be continually evaluated through observational studies involving different populations to conduct intervention programs and reduce the burden of HTN in developing countries.

## Abbreviations

BP: blood pressure
DBP: diastolic blood pressure
ESC: European Society of Cardiology
ESH: European Society of Hypertension
HTN: hypertension
LVH: left ventricle hypertrophy
NATURE-HTN: National Tunisian Registry of Hypertension
SBP: systolic blood pressure

## Data Availability

The data is the property of the Tunisian Society of Cardiology and cannot be distributed anywhere. The primary use of these data will be to describe the epidemiological profile of hypertension in Tunisia, determine the cardiovascular risk level of Tunisian hypertensive patients, and obtain an idea about the percentage of patients treated for therapeutic purposes. The planned secondary uses are to inform all physicians who may be involved in the management of Tunisian hypertensive patients (general practitioners, cardiologists, nephrologists, endocrinologists…) and to prepare a guide (in collaboration with the Ministry of Public Health), based on the outcomes of the registry, so that they can improve the level of hypertension control.
